# Characterization of Stromatolite Organic Sedimentary Structure Based on Spectral Image Fusion

**DOI:** 10.3390/s23136128

**Published:** 2023-07-03

**Authors:** Hongpeng Wang, Xinru Yan, Yingjian Xin, Peipei Fang, Yian Wang, Sicong Liu, Jianjun Jia, Liang Zhang, Xiong Wan

**Affiliations:** 1 College of Surveying and Geo-Informatics, Tongji University, Shanghai 200092, China; wanghongpeng813@163.com; 2 Key Laboratory of Space Active Opto-Electronics Technology of the Chinese Academy of Sciences, Shanghai Institute of Technical Physics, Chinese Academy of Sciences, Shanghai 200083, China; 3 Key Laboratory of Systems Health Science of Zhejiang Province, School of Life Science, Hangzhou Institute for Advanced Study, University of Chinese Academy of Sciences, Hangzhou 310024, China; 4 University of Chinese Academy of Sciences, Beijing 100049, China

**Keywords:** Raman fluorescence mapping, microscopic image, spectral image fusion, stromatolite

## Abstract

This paper evaluates the potential application of Raman baselines in characterizing organic deposition. Taking the layered sediments (Stromatolite) formed by the growth of early life on the Earth as the research object, Raman spectroscopy is an essential means to detect deep-space extraterrestrial life. Fluorescence is the main factor that interferes with Raman spectroscopy detection, which will cause the enhancement of the Raman baseline and annihilate Raman information. The paper aims to evaluate fluorescence contained in the Raman baseline and characterize organic sedimentary structure using the Raman baseline. This study achieves spectral image fusion combined with mapping technology to obtain high spatial and spectral resolution fusion images. To clarify that the fluorescence of organic matter deposition is the main factor causing Raman baseline enhancement, 5041 Raman spectra were obtained in the scanning area of 710 μm × 710 μm, and the correlation mechanism between the gray level of the light-dark layer of the detection point and the Raman baseline was compared. The spatial distribution of carbonate minerals and organic precipitations was detected by combining mapping technology. In addition, based on the BI-IHS algorithm, the spectral image fusion of Raman fluorescence mapping and reflection micrograph, polarization micrograph, and orthogonal polarization micrograph are realized, respectively. A fusion image with high spectral resolution and high spatial resolution is obtained. The results show that the Raman baseline can be used as helpful information to characterize stromatolite organic sedimentary structure.

## 1. Introduction

The search for extraterrestrial life is one of the eternal themes of deep space exploration, and it is also a major scientific problem that still needs to be solved. How to use limited means to detect the unknown world and how to use limited technology to evaluate unknown life forms are the scientific problems that human beings need to solve at this stage. Spectral detection technology is a primary means for human beings to understand the universe at this stage. The key to finding extraterrestrial life is to find direct evidence (biosignature) that life has existed. To find life outside the earth, we must understand the origin, evolution, and residual biological characteristics of life on the planet. Taking Mars as an example, NASA continues to promote the exploration of Martian life or its imprints [1,2,3], among which the most representative Curiosity and Perseverance have both carried sensitive geochemical analysis equipment to find fossil records of paleontology on Mars [4]. Fossils of Paleo-micro-organisms mainly comprise stromatolites and other sedimentary structures produced by microorganisms, which contain traces of geochemical life [5]. The oldest evidence of life on the Earth that is widely accepted and conclusive is from tiny fossils found in ~3465-million-year-old Western Australia Apex chert [6]. Scientists searched for evidence of the earliest life on the earth in the ancient continent of Greenland, which contains the oldest rocks, and found evidence of suspected life in rock 3.7 billion years ago [7]. Still, this evidence was later confirmed as an abiotic origin [8].

Raman spectroscopy is a non-invasive, non-contact molecular spectrum detection technology. Light irradiation on the object’s surface will cause elastic and inelastic scattering. The wavelength of the elastic scattering light is the same as that of the incident excitation light. The wavelength of the inelastic scattering light is usually longer than that of the excitation light. There is a nonlinear light scattering effect in this series of inelastic scattering called the Raman light scattering effect. Raman spectroscopy results from the interaction between photons and optical branch phonons [9]. Fluorescence is usually much broader than the Raman spectrum peak and appears as a slowly changing baseline, the main factor affecting Raman spectrum detection. Some materials strongly absorb photons and convert almost all absorbed photons into fluorescence—the fluorescence cross-section may be 10^10^ times larger than the Raman cross-section. Even if the concentration of these substances is shallow, the fluorescence background they produce will be stronger than the Raman spectrum produced by the unknown substance to be tested. Therefore, it is difficult to predict the composition and concentration of these trace impurities in the unknown substance to be tested. Raman spectroscopy technology is a potentially powerful means to find the life evidence of ancient microorganisms on Mars. The Raman laser spectrometer (RLS) instrument of the ExoMars rover, built by ESA in collaboration with Roscosmos, will also use Raman technology [10]. ESA conducted a series of ground tests called ExoFiT. The RLS team used a portable technology demonstrator of RLS (RAD1 system) to conduct in situ Raman detection trials in the Tabernas Desert [11] and the Atacama Desert [12]. Raman analysis detected some vibrational peaks potentially emitted by organic functional groups, thus suggesting the presence of microorganisms in the sample. In recent years, many researchers have investigated and built Raman databases on minerals that may preserve life traces, such as carbonate [13], phyllosilicate [14], and jarosite [15]. At present, the deep ultraviolet Raman fluorescence spectrometer carried by the SHERLOC instrument is looking for microbial life evidence on the surface of Mars [2], which can detect and characterize various types of natural organics [16,17,18]. So far, stromatolites have not been detected by remote or in situ analysis. However, human strategies for exploring or identifying the existence of fossils in ancient Martian sedimentary rocks are still based on the cognition of the Earth’s Precambrian life and microbial fossils and their discovery in early rock records.

Choosing a shorter or longer excitation wavelength is an effective strategy to avoid fluorescence interference. Still, a shorter excitation wavelength (deep ultraviolet spectrum) means higher optical design and processing requirements, while a longer excitation wavelength (infrared spectrum) means lower excitation efficiency and Spectral resolution. Therefore, excitation light sources with visible spectral bands still have specific application potential in practical applications. To mine more spectral information in the limited spectral data, we propose a processing procedure to evaluate organic matter using the Raman baseline and combine spectral image fusion technology to detect the spatial distribution of organic precipitation. The separation of the Raman spectrum and Raman baseline is realized through the baseline estimation and denoising with sparsity (BEADS) algorithm. First, according to the dark color characteristics of stromatolite organic precipitation, the influence of organic content on the Raman baseline is evaluated. Then, the contribution of organic fluorescence in the Raman baseline is explained. Finally, the characterization of organic precipitation structure by the Raman baseline is realized. Our work aims to evaluate the ability of new detection technology to detect stromatolite organic precipitations using the Raman baseline combined with the Bilinear interpolation- Intensity Hue Saturation (BI-IHS) algorithm spectral image fusion technology.

## 2. Materials and Methods

### 2.1. Sample Description and Preparation

**Figure 1:**
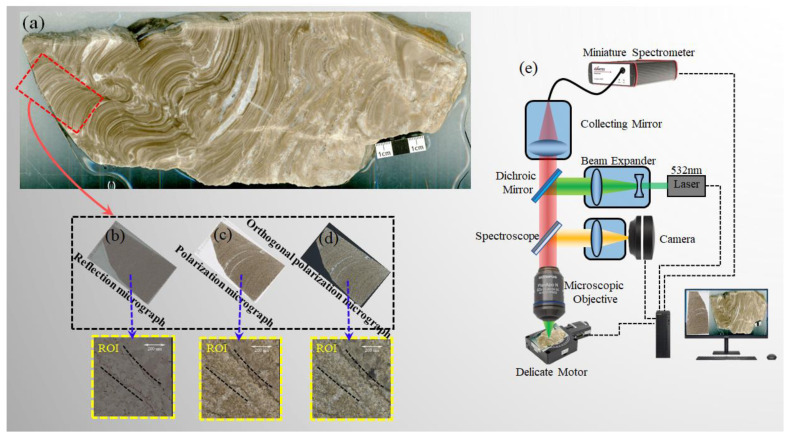
Structural diagram of stromatolite sample and experimental system (**a**) section cutting of stromatolite, (**b**) reflection micrograph, (**c**) polarization micrograph, (**d**) orthogonal polarization micrograph, (**e**) Raman mapping device diagram.

The stromatolite samples were collected near the Middle-Upper Proterozoic National Nature Reserve in Jizhou District, Tianjin, China. For protection, all sample collection activities have been prohibited in the Middle-Upper Proterozoic National Nature Reserve. Therefore, the stromatolite samples used in this paper are scattered outside the reserve, with relatively complete and well-preserved rock masses. Figure 1a shows the section of the stromatolite rock block and the area where the stromatolite slice was made (in the red box). Finally, Figure 1b–d show the reflection micrograph, the polarization micrograph, and the orthogonal polarization micrograph of the stromatolite slice, respectively. The stromatolite sample for the experiment in Raman mapping is the slice in the yellow area of Figure 1.

The production process of stromatolite samples includes cutting, polishing, gluing, slicing, and grinding. The cutting process is to cut the unweathered rock sample into an appropriate size consistent with the glass slide. The polishing process uses 600~1000 mesh silicon carbide powder to grind the cut sample to smooth its cut surface. The gluing process uses epoxy resin to polish the ground rock sample. It is adhered to frosted glass and baked at 50 °C for 8 h to solidify and harden it. The slicing process involves using a thin film cutting machine (Petro tin) to cut and grind the glued sample into approximately 120 μm thin slices. The grinding process consists in using a micrometer to calibrate the thickness of the specimen and rubbing it onto a thin lapping plate to a standard thickness of 15 μm.

### 2.2. Experimental Device

The Raman mapping device diagram is shown in Figure 1e. A green laser (Central wavelength: 532 ± 1 nm, Operating mode: CW, Maximum Power: 300 mW, Power stability: <3%, Spectral line width: <0.000001 nm) was focused on a stromatolite profile through a 100× Olympus microscope. The Dichroic Splitter (Semrock Inc., Rochester, NY, USA, LPD02-532RU-25) plays the role of light splitting, i.e., reflecting a standard 532 laser incident at 45° and transmitting the longer Raman-shifted wavelengths; Ultra long edge filter (Semrock Inc., LP03-532RE-25) can further extract weak signals, i.e., filtering the excitation light and penetrating the Raman weak signal closer to the laser line. The Raman spectrometer is a high SNR refrigerated optical fiber spectrometer (Oceanhood Technology, Shanghai, China) in a range of 150~1850 cm^−1^. The signal-to-noise ratio of the spectrometer is 1000:1. The integration time of a single Raman spectrum is 100 ms. Raman mapping scanning area 710 μm × 710 μm, focus spot diameter 1 μm, scanning step 10 μm, scanning points 71 × 71.

### 2.3. Spectral Data Pre-Processing

**Figure 2:**
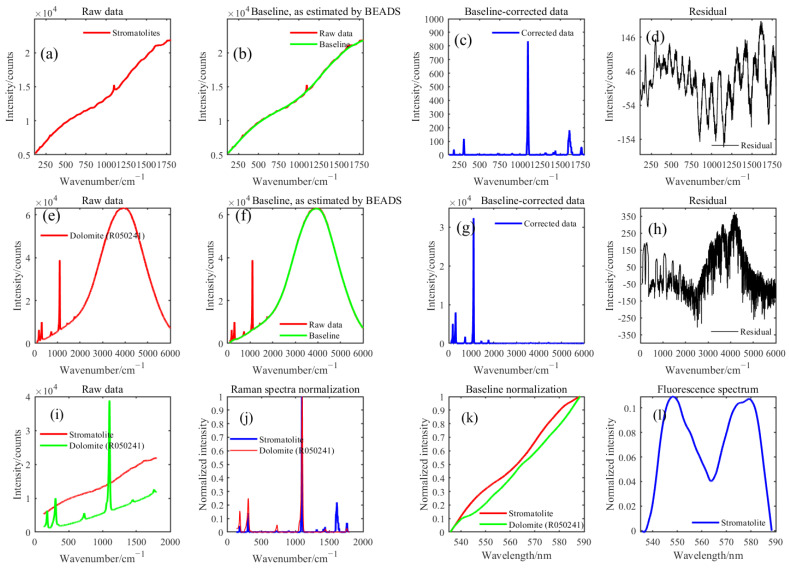
Raman baseline correction and noise reduction. (**a**) Raw Raman spectrum of stromatolite. (**b**) Raman baseline of stromatolite. (**c**) Raman spectrum of stromatolite. (**d**) residual. (**e**) Raw Raman spectrum of dolomite. (**f**) Raman baseline of dolomite. (**g**) Raman spectrum of dolomite. (**h**) residual. (**i**) Raw Raman spectra of stromatolite and dolomite. (**j**) Raman spectra of stromatolite and dolomite. (**k**) Raman baselines of stromatolite and dolomite. (**l**) Raman baseline difference between stromatolite and dolomite.

Raman spectroscopy is a nondestructive detection technology which is easily affected by the environmental background light, fluorescence, and dark current and has noticeable baseline drift. Fortunately, Raman spectrum signals with the sparse spectral peaks and low pass filtering characteristics of baseline. The BEADS algorithm is suitable for solving the problem of baseline correction and noise reduction of one-dimensional signals. The algorithm models the baseline as a low-pass signal, while the spectral peak is sparse and has sparse derivatives. When a low-order polynomial is unsuitable for baseline modeling, the BEADS algorithm is superior to the polynomial modeling method. It has the characteristics of high efficiency, fast iterative convergence, and stability [19]. BEADS has been successfully applied to baseline correction of Raman spectra and spectral data denoising [20,21,22,23]. The cut-off frequency Fc, filtering order parameter D, and asymmetry parameter R of the BEADS algorithm used in this paper were 0.05, 1.00, and 6.00, respectively. The area normalization of baseline-corrected and noise-reduced data can reduce the influence of laser-focusing energy difference, detection distance difference, and other factors on the spectrum. Figure 2c is the Raman spectrum of the white stripe part of the stromatolite slice. By retrieving the RRUFF Project website’s Raman Spectrum Database, we matched the Raman spectrum of dolomite, as shown in Figure 2e. After undergoing the same parameter BEADS algorithm preprocessing, the baseline, Raman signal, and residual of the Raman spectrum of dolomite are shown in Figure 2f–h. Figure 2j shows the Raman spectra comparison of the two substances, Figure 2k shows the Raman baseline comparison of the two substances, and Figure 2l shows the Raman baseline differences between the two substances, indicating that baseline of stromatolites exhibits unique spectroscopy characteristics compared to dolomite.

### 2.4. Algorithm

**Figure 3:**
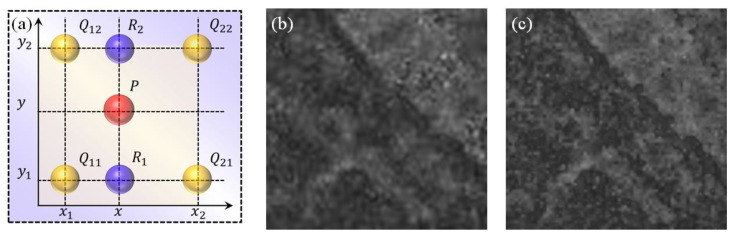
Basic principle of the BI algorithm. (**a**) Basic principle of Bilinear interpolation. (**b**) Original image. (**c**) Image after Bilinear interpolation.

Regarding data processing, Matlab R2020a (Massachusetts Institute of Technology, Natick, MA, USA) is used for chemical analysis and scientific drawing, respectively. The schematic diagram in the article is drawn by Microsoft Office Professional Enhanced Edition 2019 PowerPoint (Microsoft, Redmond, WA, USA), as shown in Figure 1 and Figure 3.

#### BI-IHS Model

Bilinear interpolation (BI) is often used to improve image quality after performing spatial transformation operations [24]. For example, suppose we want to find the value of the unknown function at the P point (*x*, *y*). It is assumed that we know the value of f at the four points $Q_{11}=\left(x_{1},y_{1}\right)$, $Q_{12}=\left(x_{1},y_{2}\right)$, $Q_{21}=\left(x_{2},y_{1}\right)$, $Q_{22}=\left(x_{2},y_{2}\right)$, as shown in Figure 3a. We first perform linear interpolation in the x-direction:

$$\begin{array}{c} f\left(R_{1}\right)=f\left(x,y_{1}\right)\approx \frac{x_{2}-x}{x_{2}-x_{1}}f\left(Q_{11}\right)+\frac{x-x_{1}}{x_{2}-x_{1}}f\left(Q_{21}\right)\\ f\left(R_{2}\right)=f\left(x,y_{2}\right)\approx \frac{x_{2}-x}{x_{2}-x_{1}}f\left(Q_{12}\right)+\frac{x-x_{1}}{x_{2}-x_{1}}f\left(Q_{22}\right) \end{array}$$

Then, we perform linear interpolation in the y-direction:

(1) $$\begin{array}{l} f\left(x,y\right)\approx \frac{y_{2}-y}{y_{2}-y_{1}}f\left(R_{1}\right)+\frac{y-y_{1}}{y_{2}-y_{1}}f(R_{2})\\ =\frac{y_{2}-y}{y_{2}-y_{1}}\left(\frac{x_{2}-x}{x_{2}-x_{1}}f\left(Q_{11}\right)+\frac{x-x_{1}}{x_{2}-x_{1}}f\left(Q_{21}\right)\right)+\frac{y-y_{1}}{y_{2}-y_{1}}\left(\frac{x_{2}-x}{x_{2}-x_{1}}f\left(Q_{12}\right)+\frac{x-x_{1}}{x_{2}-x_{1}}f\left(Q_{22}\right)\right)\\ =\frac{1}{\left(x_{2}-x_{1}\right)\left(y_{2}-y_{1}\right)}\left(f\left(Q_{11}\right)\left(x_{2}-x\right)\left(y_{2}-y\right)+f\left(Q_{21}\right)\left(x-x_{1}\right)\left(y_{2}-y\right)+f\left(Q_{12}\right)\left(x_{2}-x\right)\left(y-y_{1}\right)+f\left(Q_{22}\right)\left(x-x_{1}\right)\left(y-y_{1}\right)\right)\\ =\frac{1}{\left(x_{2}-x_{1}\right)\left(y_{2}-y_{1}\right)}\left[x_{2}-xx-x_{1}\right]\left[\begin{array}{cc} f\left(Q_{11}\right) & f\left(Q_{12}\right)\\ f\left(Q_{21}\right) & f\left(Q_{22}\right) \end{array}\right]\left[\begin{array}{c} y_{2}-y\\ y-y_{1} \end{array}\right] \end{array}$$

The Intensity-Hue-Saturation (IHS) algorithm is a mature spatial transformation algorithm that was developed very early in the history of image fusion technology [25,26,27,28]. This work is based on the IHS algorithm to achieve the spectral image fusion of micrograph and Raman fluorescence mapping. The core of this method is to convert the multispectral RGB space into an IHS space based on the IHS transform, where I represents intensity, H represents hue, and S represents saturation. The brightness component I mainly reflects the spatial information of the multispectral image. In contrast, the hue component H and saturation component S mainly reflect the spectral information of the multispectral image. Due to the slight correlation between spectral information and the I component, high-resolution images replace this component and perform inverse transformations with the original H and S components, thereby improving the spatial resolution of multispectral images and maintaining the original spectral information.

**Figure 4:**
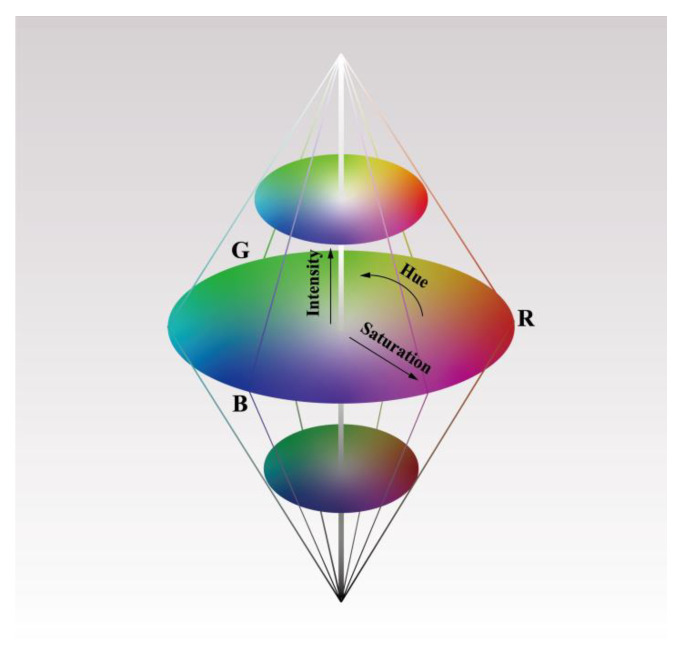
IHS spatial diagram.

The IHS color space can be described by a conic space model, as shown in Figure 4. I, H, and S are height, azimuth, and radial distance. The I component changes along the axis from black at the bottom to white at the top; H and S are a pair of polar coordinates in a plane perpendicular to I; H is represented by angle, stipulating that 0° is red, 120° is green, and 240° is blue, and then 0~240° covers the color of all visible spectrum; S is the length of the radius from the origin of the chroma ring to the color point, with a saturation of 0 for the center of the circle and 1 for the circumference.

**Figure 5:**
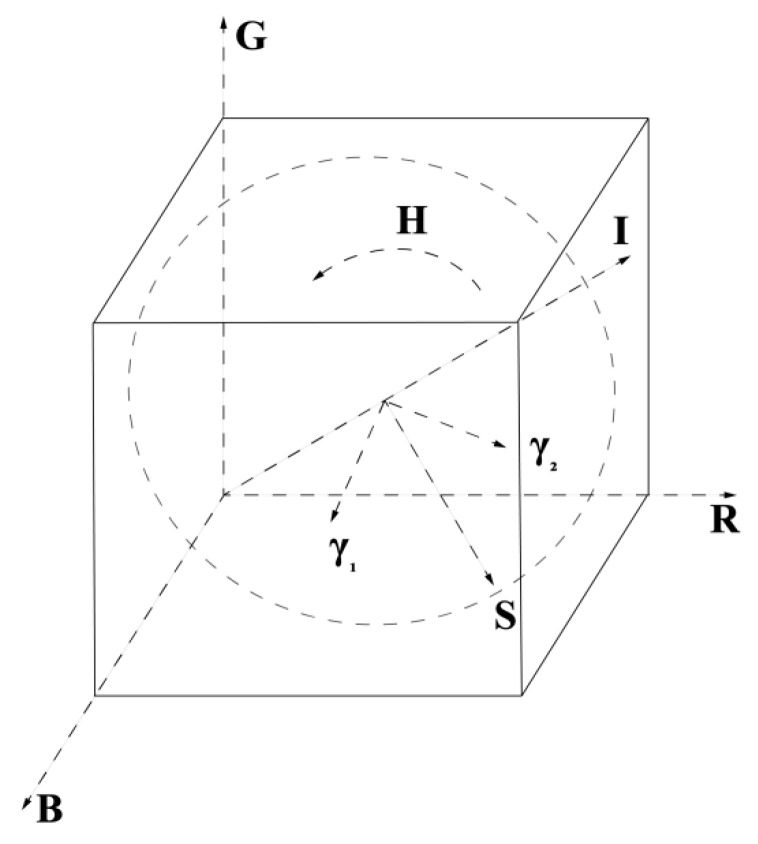
Spatial relationship between RGB and IHS.

The diagonal of the RGB color cube coincides with the central axis of the IHS color cylindricum, as shown in Figure 5. The IHS value of the image has the following relationship with RGB:

$$H=\left\{\begin{array}{c} \theta \\ 360-\theta \end{array}\begin{array}{c} \;B\leq G\\ \;B>G \end{array}\right.$$

with

$$\theta =\mathrm{ar}\;\cos\left\{\frac{\left[\left(R-G\right)+\left(R-B\right)\right]/2}{\sqrt{{\left(R-G\right)}^{2}+\left(R-B\right)\times \left(G-B\right)}}\right\}$$

The saturation component is given by:

$$S=1-\frac{3\;\min\left(R,G,B\right)}{R+G+B}$$

Finally, the intensity component is given by:

$$I=\frac{1}{3}\times \left(R+G+B\right)$$

The fusion process of the three bands of multispectral images is as follows:

Step 1: Resample the low spatial resolution multispectral image to the panchromatic image size, then conduct the positive transformation of the resampled multispectral image from RGB to IHS space. Here, the bilinear interpolation method is used for resampling processing.

$$\left(\begin{array}{c} I\\ \gamma_{1}\\ \gamma_{2} \end{array}\right)=\left(\begin{array}{ccc} \frac{1}{3} & \frac{1}{3} & \frac{1}{3}\\ -\frac{\sqrt{2}}{6} & -\frac{\sqrt{2}}{6} & \frac{2\sqrt{2}}{6}\\ \frac{1}{\sqrt{2}} & \frac{1}{\sqrt{2}} & 0 \end{array}\right)\left(\begin{array}{c} \tilde{y_{1}}\\ \tilde{y_{2}}\\ \tilde{y_{3}} \end{array}\right)$$

Among them, $\tilde{y_{1}}$, $\tilde{y_{2}}$, and $\tilde{y_{3}}$ represent the R, G, and B bands of the resampled multispectral image, where I represents the brightness component and $\gamma_{1}$ and $\gamma_{2}$ are cartesian coordinates corresponding to the polar coordinates of H and S, respectively. As shown in Figure 5, $H=\tan^{-1}\left(\frac{\gamma_{2}}{\gamma_{1}}\right)$ and $S=\sqrt{\gamma_{1}^{2}+\gamma_{2}^{2}}$ represent the hue component and saturation component, respectively.

Step 2: Moment matching is performed between the panchromatic image f and the brightness I component so that it has the same mean and variance as the replaced I component. The purpose is to reduce spectral distortion in the fused image, which can be expressed as:

$$f\prime \left(i,j\right)=\frac{\sigma_{I}}{\sigma_{f}}\times \left(f\left(i,j\right)-\;f\;¯\right)\;I\;¯$$

Among them, $f\left(i,j\right)$ represents the pixel value of the panchromatic image at position (i, j), $f\prime \left(i,j\right)$ represents the pixel value of the panchromatic image at position (i, j) after moment matching,  f ¯ represents the mean of the panchromatic image,  I ¯ represents the mean of the brightness component, $\sigma_{I}$ represents the standard deviation of the brightness component I, and $\sigma_{f}$ represents the standard deviation of the panchromatic image.

Step 3: Replace the brightness component I with the panchromatic image after moment matching, and perform IHS inverse transformation with the hue H and saturation S components to obtain a high spatial resolution multispectral image, which can be expressed as:

$$\left(\begin{array}{c} x_{1}\\ x_{2}\\ x_{3} \end{array}\right)=\left(\begin{array}{ccc} 1 & -\frac{1}{\sqrt{2}} & \frac{1}{\sqrt{2}}\\ 1 & -\frac{1}{\sqrt{2}} & -\frac{1}{\sqrt{2}}\\ 1 & \sqrt{2} & 0 \end{array}\right)\left(\begin{array}{c} z\prime \\ v_{1}\\ v_{2} \end{array}\right)$$

Among them, $x_{1}$, $x_{2}$ and $x_{3}$ represent the three bands of the fused image.

## 3. Results

### 3.1. The Raman Baseline—Fluorescence

**Figure 6:**
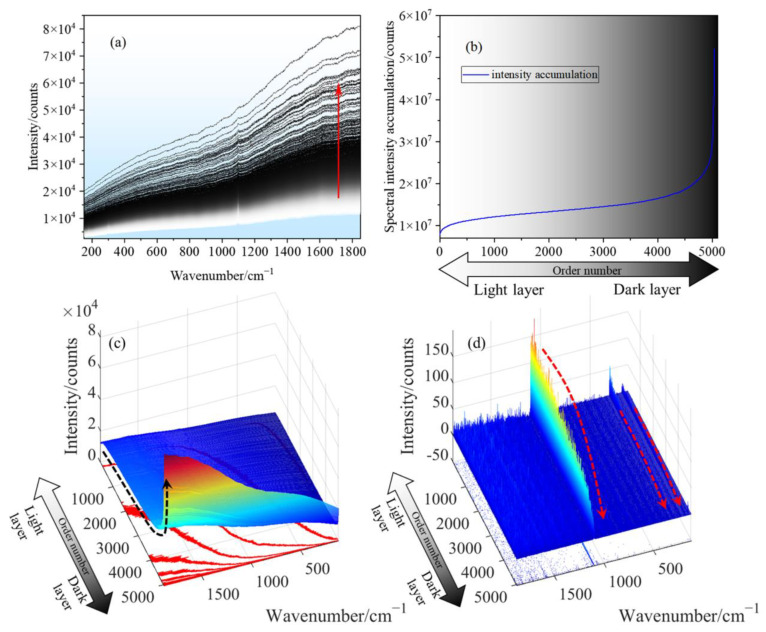
Spectral characteristics of stromatolite light and dark layers. (**a**) Raw Raman spectra corresponding to the gray level of scanning points microscopic image. (**b**) Curve of spectral intensity accumulation changing with the gray scale of the scanning point. (**c**) 3D surface drawing of Raman baseline changes with organic precipitation. (**d**) 3D surface drawing of Raman spectra changes with organic precipitation.

Stromatolite layered deposit, mainly of limestone, is formed by the growth of blue-green algae (primitive one-celled organisms). It is well known that the parts with high organic content in stromatolites are dark in color. In contrast, the regions with white color are mainly composed of sand and less organic content, and the structures are usually characterized by thin, alternating light and dark layers. The original spectra in the Raman scanning area are arranged according to the gray scale of the microscopic image corresponding to the scanning point, as shown in Figure 6a. Light layers correspond to the spectra of white lines, and dark correspond to the spectra of black lines. It can be found that the spectral intensity corresponding to dark layers is higher, and the spectral intensity accumulation increases exponentially with the increase in organic precipitation (dark layers), as shown in Figure 6b.

The source of the Raman baseline is complex, related to the material’s physical property (the morphology of the sample) and chemical property (photoluminescent material), and other factors (ambient light, temperature fluctuations, and noise). With the same chemical composition of materials, the samples in the fine powder state are more likely to produce a strong, broad spectral background. In contrast, the models with compressed particles have better Raman spectral signals and weak spectral backgrounds. The main reason for this phenomenon is that the diffuse reflection of a fine powder sample will cause intense stray light rather than photoluminescence [29]. From the perspective of the physical properties of materials, the stromatolites studied in this paper and the matched dolomite (RRUFFID: R050241) samples in the database are solid, and the Raman baseline caused by their physical properties is similar. Therefore, this paper mainly attributed the Raman baseline difference of the stromatolites and dolomite to the accretionary organosedimentary structure.

The spectral preprocessing based on the BEADS algorithm can realize the separation of the Raman spectrum and baseline. Figure 6c,d shows the three-dimensional surface of the Raman baseline and Raman spectrum of stromatolites as a function of image gray. Figure 6c shows that the Raman baseline of the dark layer increases exponentially. The essential factor that causes this exponential increase is the strong fluorescence signal generated by organic precipitation, and this strong fluorescence will annihilate the Raman signal. Therefore, in Figure 6d, the Raman signal will weaken or disappear with the transition from the bright to the dark layer. Based on the above analysis, the organic precipitation of the stromatolite biosphere showed strong fluorescence characteristics in this experiment. The fluorescence generated by organic precipitation played a decisive role in the Raman baseline.

**Figure 7:**
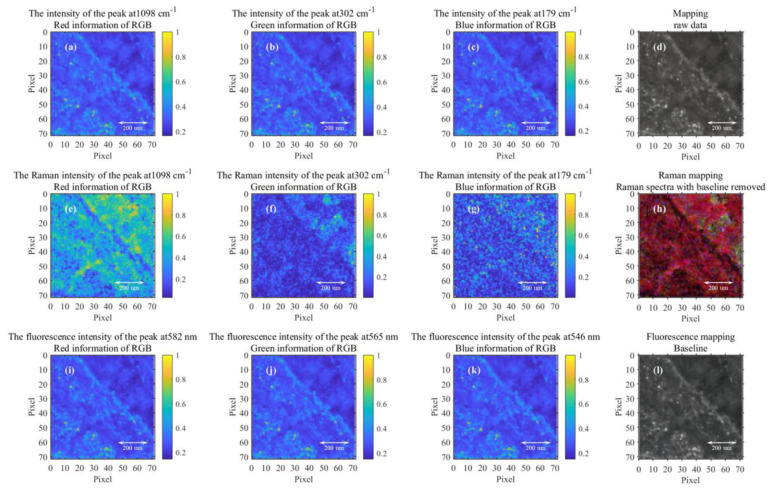
Raman mapping and fluorescence mapping. (**a**–**c**) The intensity mapping of the original Raman spectrum at 1098, 302, and 179 cm^−1^, respectively. (**d**) The pseudo-color image synthesized in (**a**–**c**). (**e**–**g**) The intensity mapping of the Raman spectrum after data preprocessing at 1098, 302, and 179 cm^−1^, respectively. (**h**) The pseudo-color image synthesized in (**e**–**g**). (**i**–**k**) The intensity mapping of the fluorescence spectrum at 582, 565, and 546 nm, respectively. (**l**) The pseudo-color image synthesized in (**i**–**k**).

The Raman mapping scanning area of the rock mass is shown in Figure 1. The yellow area (about 710 μm × 710 μm) is the optical microscopic image of the region of interest (ROI). Under the focusing spot of 1 μm and scanning step of 10 μm, we can obtain the Raman spectra of 5041 points in this area. Three prominent Raman peaks of carbonate minerals are in the Raman spectrum after baseline removal, which at 1098, 302, and 179 cm^−1^ are generated by the internal mode (v1), lattice modes (L), and lattice modes (T), respectively [30]. This paper compares the mapping constructed by the raw Raman spectra, the Raman spectra removed baseline, and the fluorescence spectra, as shown in Figure 5. If the baseline is not removed, even if the intensity of the Raman spectral peak is selected for mapping, the spectral information of the Raman spectrum cannot be reflected. Instead, the mapping only reflects the information on the fluorescence. Raman mapping after baseline removal reflects the spatial distribution characteristics of carbonate. Fluorescent mapping at different wavelength positions reflects the same information as the original Raman spectrum mapping, as shown in Figure 7d,l. Raman mapping represents the spatial distribution of carbonate minerals, while the fluorescence spectrum represents the “imprint”—organic residue kept by microbial activities.

### 3.2. The Fusion of Microscopic Image and Raman Fluorescence Mapping

**Figure 8:**
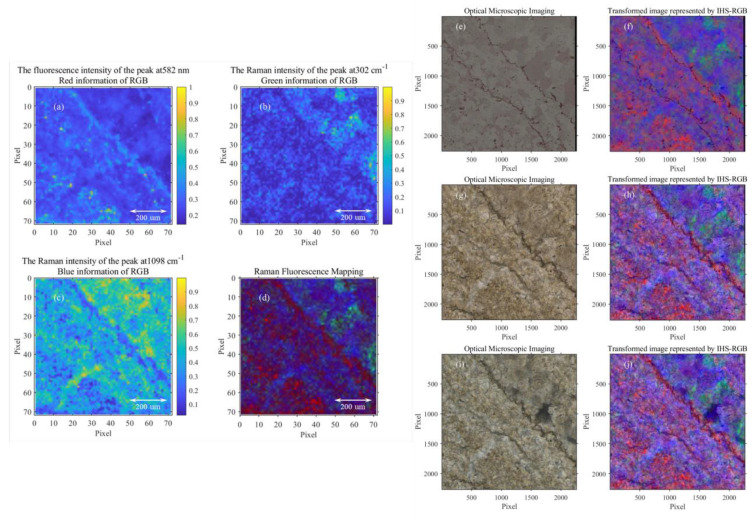
The fusion of Raman mapping and microscopic image. (**a**) The intensity mapping of fluorescence spectrum at 582 nm. (**b**,**c**) Raman spectrum intensity mapping at 302, 1098 cm^−1^. (**d**) The pseudo-color image synthesized in (**a**–**c**). (**e**) The reflection micrograph of the slice. (**f**) IHS fusion map of (**d**,**e**). (**g**) The polarization of the slice. (**h**) IHS fusion map of (**d**,**g**). (**i**) The orthogonal polarization micrograph of the slice. (**j**) IHS fusion map of (**d**,**i**).

Although the BI algorithm has improved the mapping quality to a certain extent, the spatial resolution is not up to that of optical images. However, spectral image fusion can provide spatial resolution and spectral resolution. This part mainly uses the IHS algorithm to realize the fusion of microscopic images and Raman fluorescence mapping, as shown in Figure 8f–j. The experimental results show that the fluorescence mapping results are highly consistent with the spatial distribution of organic precipitation, which shows the potential of fluorescence mapping in characterizing organic precipitation.

## 4. Discussion

### 4.1. Astrobiology Significance of Stromatolites

Stromatolites are biological sedimentary structures formed by ancient microorganisms (mainly blue-green algae) that absorb, precipitate minerals, or capture mineral particles through growth and metabolic activities [31]. Therefore, the formation of stromatolites is closely related to the life activities of stromatolite-forming microorganisms, their ancient environment, and climate. The dynamic action of microorganisms, coupled with periodic changes in their growth and sedimentation (such as day and night, seasonal, and annual changes), distinguishes them from other non-biological geological sedimentary structures, forming morphological with unique structure features from the inside out. For the widely distributed Precambrian stromatolites, in addition to morphological studies, an in-depth discussion of microorganisms that produce stromatolites and their biological sedimentation activities is an essential part of the study of the evolution and preservation mechanisms of early planetary life, especially the study of the control mechanism of the basic dark layer structure of stromatolites by biological factors, that is, the micro control of biological factors on stromatolites.

Currently, the strategy for searching for extraterrestrial life is mainly to take the Precambrian microbial fossils of the Earth as the model and to study the preservation potential and detection methods of potential microbial fossil life on Mars by analogy based on spectral technology. Stromatolites record essential clues to the existence of early life on the Earth and have extremely typical macro characteristics and microstructures. Furthermore, Mars may also have had environmental conditions for stromatolites to “grow” in its ancient history. The discovery of stromatolite-like minerals is evidence of extraterrestrial life’s existence and a vital search target for Martian rock sampling back to Earth in the future. Therefore, this study has reference value for searching for extraterrestrial stromatolite-like minerals.

### 4.2. Relationship between Stromatolites and Sedimentary Facies

**Figure 9:**
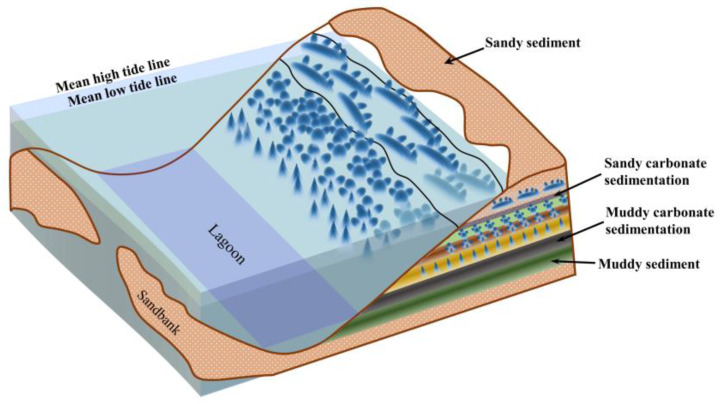
Schematic diagram of the relationship between the types of stromatolites and sedimentary facies in the sampling area (modified from Zhu, S., 1993 [32]).

In the general environment where stromatolites are formed, they are mainly found in the upper section of clear water carbonate deposits. In contrast, it is not easy to find the presence of stromatolites in the muddy lower area of turbid water carbonate deposits. Therefore, the primary environmental conditions for forming stromatolites are carbonate-rich (or similar salts) and relatively clear media conditions. Figure 9 shows a schematic diagram of the relationship between stromatolite types and sedimentary facies in the sampling area. The sedimentary environment has an important impact on the formation of stromatolites, forming sedimentary sequences that become shallower upwards through regression sedimentation, characterized by different types of production from bottom to top. The relationship between the types of stromatolites and sedimentary facies is manifested as the convexity of the basic units of stromatolites and is related to the water depth of the growth environment. The deeper the depth, the greater the convexity, while the shallower the depth, the smaller the convexity; there is a significant correlation between the branching of stromatolites and the energy of water bodies. Stromatolites with simple or no branching are generally formed in low-energy water environments, while complex branching stromatolites are usually constructed in high-energy water environments; the thickness of the basic layer is related to the sedimentation rate, and the thicker the basic layer, the faster the sedimentation rate.

### 4.3. Scientific Research Value of Searching for Stromatolites on Planets

So far, the Earth is the only planet known to have given birth to life, but compared to the long process of cosmic evolution, many extraterrestrial planets have the conditions and timing to give birth to life. Although the current environment may be extremely harsh, such as modern Mars, it may have been a warm and humid climate suitable for life for a considerable period. By analogy with the evolution process and preservation mechanism of life on the Earth, stromatolites are an essential research object in paleontology and sedimentology, the preferred research object for the analogical study of extraterrestrial life, and also a unique biological sedimentary structure that is still forming from the pre-Cambrian period to the present, with a wide variety, a large number, and strong representativeness. Researching the morphology, sedimentology, and paleontology of stromatolites on the Earth has essential reference value for searching for extraterrestrial life. The Deep Ultraviolet Raman Spectrometer (SHERLOC) carried by the Persevere Mars rover uses a 248.6 nm excitation source to avoid interference from fluorescence signals on the Raman spectrum [16,18]. However, the ExoMars Rover of the delayed launch also carries a Raman spectrometer with a 532 nm continuous laser as the excitation source [33], which may exhibit fluorescence Raman superposition interference at this wavelength. Therefore, this article evaluates the feasibility of extracting Raman spectra of stromatolites and using redundant signals (baseline) under fluorescence interference.

### 4.4. Potential Applications and Limitations in Other Fields

In addition, this scheme provides an effective method for mineral exploration in geological or environmental sciences and other fields of the Earth. The weak Raman spectral signal has always been a challenge in this field. Suppose the influence of factors such as ambient light, temperature fluctuations, and noise can be excluded. In that case, the Raman baseline generated by physical and chemical properties is essential in analyzing mineral properties. However, this technology also has limitations, as the conclusion that the main factor causing Raman baseline is attributed to organic matter deposition is supported by micrographs. In the practical application of deep space exploration, producing laser thin films on-site is almost impossible. Therefore, many ground simulation experiments are needed to comprehensively evaluate the impact of physical and chemical properties on the Raman baseline, especially for the Raman baseline problem caused by organic matter deposition in different minerals.

## 5. Conclusions

In this work, we proposed a method to realize a spectral image fusion map with high spatial resolution and high spectral resolution of stromatolite microstructure. We evaluated the feasibility of the Raman baseline as a fluorescence spectrum of organic precipitation. To improve the utilization of spectral data, the BEADS algorithm is used to separate the Raman spectrum from the Raman baseline, and the corresponding relationship between the Raman baseline and the stromatolite light and dark layers is analyzed. It is found that the fluorescence of organic precipitation is the main factor that causes the enhancement of the Raman baseline index. Therefore, the spectral image fusion of spectral mapping and microscopic images is realized based on the BI-IHS algorithm, and the spatial distribution of carbonate minerals and organic precipitations are obtained, respectively.

In conclusion, our research shows that the Raman baseline is not necessarily redundant information. It may contain the fluorescence of organic sediments. Under the condition that the kerogen Raman spectrum peak is not detected, the spatial distribution of organic residues can be seen by combining the fluorescence information in the Raman baseline with the mapping technology.

## Data Availability

Data underlying the results presented in this paper are not publicly available at this time but may be obtained from the authors upon reasonable request.
